# Species-Specificity of Transcriptional Regulation and the Response to Lipopolysaccharide in Mammalian Macrophages

**DOI:** 10.3389/fcell.2020.00661

**Published:** 2020-07-21

**Authors:** Stephen J. Bush, Mary E. B. McCulloch, Zofia M. Lisowski, Charity Muriuki, Emily L. Clark, Rachel Young, Clare Pridans, James G. D. Prendergast, Kim M. Summers, David A. Hume

**Affiliations:** ^1^Nuffield Department of Clinical Medicine, John Radcliffe Hospital, University of Oxford, Oxford, United Kingdom; ^2^The Roslin Institute, The University of Edinburgh, Edinburgh, United Kingdom; ^3^Centre for Inflammation Research, The University of Edinburgh, Edinburgh, United Kingdom; ^4^Simons Initiative for the Developing Brain, Centre for Discovery Brain Sciences, The University of Edinburgh, Edinburgh, United Kingdom; ^5^Mater Research Institute-University of Queensland, Translational Research Institute, Woolloongabba, QLD, Australia

**Keywords:** transcriptome, macrophage, LPS, feedback, network, conservation, species

## Abstract

Mammalian macrophages differ in their basal gene expression profiles and response to the toll-like receptor 4 (TLR4) agonist, lipopolysaccharide (LPS). In human macrophages, LPS elicits a temporal cascade of transient gene expression including feed forward activators and feedback regulators that limit the response. Here we present a transcriptional network analysis of the response of sheep bone marrow-derived macrophages (BMDM) to LPS based upon RNA-seq at 0, 2, 4, 7, and 24 h post-stimulation. The analysis reveals a conserved transcription factor network with humans, and rapid induction of feedback regulators that constrain the response at every level. The gene expression profiles of sheep BMDM at 0 and 7 h post LPS addition were compared to similar data obtained from goat, cow, water buffalo, horse, pig, mouse and rat BMDM. This comparison was based upon identification of 8,200 genes annotated in all species and detected at >10TPM in at least one sample. Analysis of expression of transcription factors revealed a conserved transcriptional millieu associated with macrophage differentiation and LPS response. The largest co-expression clusters, including genes encoding cell surface receptors, endosome–lysosome components and secretory activity, were also expressed in all species and the combined dataset defines a macrophage functional transcriptome. All of the large animals differed from rodents in lacking inducible expression of genes involved in arginine metabolism and nitric oxide production. Instead, they expressed inducible transporters and enzymes of tryptophan and kynurenine metabolism. BMDM from all species expressed high levels of transcripts encoding transporters and enzymes involved in glutamine metabolism suggesting that glutamine is a major metabolic fuel. We identify and discuss transcripts that were uniquely expressed or regulated in rodents compared to large animals including *ACOD1*, CXC and CC chemokines, *CD163*, *CLEC4E*, *CPM*, *CSF1*, *CSF2*, *CTSK*, *MARCO*, *MMP9*, *SLC2A3*, *SLC7A7*, and *SUCNR1*. Conversely, the data confirm the conserved regulation of multiple transcripts for which there is limited functional data from mouse models and knockouts. The data provide a resource for functional annotation and interpretation of loci involved in susceptibility to infectious and inflammatory disease in humans and large animal species.

## Introduction

Macrophages and related members of the mononuclear phagocyte system (MPS) have many trophic roles in development and homeostasis and are the first line of defense against potential pathogens (Hume et al., 2019; Guilliams et al., 2020). The survival, proliferation and differentiation of macrophages depends upon signaling via the macrophage colony stimulating factor receptor (CSF1R), which mediates signals from colony stimulating factor 1 (CSF1; also known as macrophage colony stimulating factor) or interleukin 34 (IL34) (Stanley and Chitu, 2014; Hume et al., 2020). In response to pathogen challenge, resident macrophages are activated to produce cytokines and chemokines that drive recruitment of neutrophils and inflammatory monocytes. The activation of macrophages is mediated through pattern recognition receptors that bind to pathogen-associated molecules (Brubaker et al., 2015). The archetypal pattern recognition receptor is TLR4, which, with the coreceptor MD-2, recognizes endotoxin or lipopolysaccharide (LPS), a major constituent of the cell wall of Gram-negative organisms (Marongiu et al., 2019). TLR4 ligation initiates the up and down regulation of thousands of transcripts, including hundreds of transcription factors (Kaikkonen et al., 2013; Baillie et al., 2017). Many of the induced genes are required for defense against pathogens, but they are also responsible for symptoms such as fever and much of the pathology. Feedback control by numerous negative regulators is therefore required to ensure that the response to pathogens is limited and appropriate (Wells et al., 2005; Kondo et al., 2012).

Many studies of LPS signaling *in vitro* have used bone marrow-derived macrophages (BMDM), cells grown from bone marrow in the presence of CSF1, or monocyte-derived macrophages (MDM), matured from blood in the presence of CSF1. Previous network analysis of the time course of human MDM response to LPS revealed a sequential cascade of transient induction of feed forward and feedback regulators (Baillie et al., 2017). Not surprisingly, given the central role of macrophages in innate immunity, there are differences in the response to LPS of mouse and human macrophages grown in CSF1 (Schroder et al., 2012). The response to the endogenous anti-inflammatory agonists, glucocorticoids, is even more divergent, associated with gain and loss of functional glucocorticoid receptor binding sites in the genome (Jubb et al., 2016). Comparative analysis in the pig indicated that BMDM and monocyte-derived macrophages grown in CSF1 have very similar gene expression profiles. Both basal and LPS-induced gene expression profiles in pig were more similar to humans than were those in mice (Kapetanovic et al., 2012, 2013).

Nitric oxide (NO) production from arginine by NOS2 is a significant component of host defense in rodent species that is not conserved in large animals. Macrophages from humans and pigs do not produce NO in response to LPS and the enhancer elements involved in *NOS2* induction are not conserved in rodents (Kapetanovic et al., 2012; Schroder et al., 2012; Karagianni et al., 2017; Young et al., 2018). To further document the species specificity of regulated arginine metabolism we cultured BMDM from sheep, goat, cattle, water buffalo, pig, horse, and rat and incubated them with or without LPS. RNA-seq analysis of these populations revealed variation in arginine metabolism amongst the species including a divergence between bovids (cattle and water buffalo) and small ruminants (sheep and goats) (Young et al., 2018). In a separate study, the same primary RNA-seq data were used to document evolution and expression of the *ADGRE1* gene, encoding F4/80, a widely used marker for macrophage biology in mouse (Waddell et al., 2018). In these studies, the LPS response was analyzed at a single timepoint in each species (7 h) chosen to coincide with maximal induction of transcripts encoding inflammatory cytokines in human monocyte-derived macrophages (Baillie et al., 2017).

Macrophage immunometabolism is a burgeoning field based upon the view that metabolic requirements change with functional polarization (Hotamisligil, 2017; Castegna et al., 2020; Ryan and O’Neill, 2020). Published studies have focused on regulation of the tricarboxylic acid (TCA) cycle and accumulation of intermediates such as itaconate, succinate, and ketoglutarate as signaling molecules (Ryan and O’Neill, 2020). Like the NOS2 pathway, much of the evidence for roles of metabolic intermediates and enzymes in macrophage activation/polarization derives from *in vitro* studies of inbred mice, and at least some of the effects of LPS on mitochondrial function are mediated by endogenous NO (Van den Bossche et al., 2017). Itaconate, produced through the induction of the enzyme ACOD1, which diverts citrate from the TCA cycle, has been associated with anti-inflammatory roles (Mills et al., 2018). Similarly, a recent study of mice described the biosynthesis of anti-inflammatory fatty acids late in the LPS response as part of the feedback control network described above (Oishi et al., 2017). It is unclear how many of the findings can be translated to humans or other species.

The domestic sheep, like the pig, is an important livestock species, and also used extensively as a model in biomedical research. BMDM have previously been grown from sheep bone marrow in CSF1 and were shown to be responsive to LPS (Francey et al., 1992a, b). Like pig and human macrophages, sheep (and goat) macrophages make no detectable NO in response to LPS (Jungi et al., 1996; Young et al., 2018). However, immunometabolism in sheep, a ruminant species, is potentially quite different from monogastric species such as humans and pigs. Mouse and rat macrophages have been shown to metabolize glutamine at a rapid rate (Curi et al., 2017). Glutamine metabolism in macrophages is regulated and inhibition of glutamine synthetase (GSS), which produces glutamine from glutamate, was shown to alter the polarization state of mouse macrophages (Jha et al., 2015; Palmieri et al., 2017). The circulating glutamine concentration in ruminants is three–fivefold lower than in monogastric species, due to a low glutamine synthetase capacity, and glutamine is not the predominant respiratory fuel for the intestine (Meijer et al., 1993). The sheep, as a ruminant, has high circulating levels of fermentative by-products, primarily volatile fatty acids (propionate, acetate, and butyrate), which are utilized within the liver for gluconeogenesis (Danfaer et al., 1995). Aside from acting as fuels, free fatty acids may be recognized by a large family of G protein coupled receptors (Kimura et al., 2020).

To extend our knowledge of the diversification of macrophage function amongst species, we have generated a time course of the transcriptomic response of sheep BMDM to LPS. Detailed analysis of this time course reveals those components that distinguish sheep from human macrophages. Comparative analysis with RNA-seq data from other species, including humans, is compromised by incomplete annotation, inconsistent naming and ambiguous orthology relationships (especially in multigene families). To enable such a comparison of sheep RNA-seq data with previously generated RNA-seq data for BMDM from goat, cow, water buffalo, horse, pig, and rat, and public domain data for two mouse species, we undertook an annotation effort to identify >8,000 macrophage-expressed genes that are clear orthologs between the species. We present a resource for functional annotation and interpretation of loci involved in susceptibility to infectious and inflammatory disease in humans and large animal species.

## Materials and Methods

### Data Generation and Analysis

The protocol for the generation of bone marrow-derived macrophages (BMDM) in recombinant CSF1 was originally developed for pigs (Kapetanovic et al., 2012, 2013). Full details of the animals and the protocol for generation and activation of BMDM from sheep marrow are included with our high resolution sheep transcriptomic atlas where data for 3 male and 3 female cross-bred adult animals were originally described (Clark et al., 2017). The mRNA sequencing libraries generated for all time points were prepared using the Illumina TruSeq stranded mRNA library preparation kit (Illumina; Part: 15031047 Revision E) and sequenced to a depth of >25 million 125 bp paired-end reads per sample as described (Clark et al., 2017).

RNA-seq libraries for control and LPS-stimulated BMDM from additional species were downloaded from the European Nucleotide Archive (ENA). Details of all of the accessions are provided in Supplementary Table S1. This comparative dataset comprises eight additional species: buffalo, goat, cow, horse, mouse (both *Mus musculus* and its outbred relative, *Mus spretus*), pig and rat, as well as the sheep. For all species except mouse, the agonist used was *Salmonella minnesota* Re595 LPS, which is a pure TLR4 agonist (Young et al., 2018). In the case of the two mouse species, the agonist used was KLA (Kdo2-lipid A) the active core of LPS (Link et al., 2018) and samples were obtained after 6 h.

While publicly sourced RNA-seq libraries can differ both in preparation and sequencing methods, it is possible to process their data with a common normalization, producing comparable expression level estimates (Summers et al., 2019). Central to this process is reducing the distorting effects of differential sampling depth. To do so, each library was randomly down-sampled to a depth of 10 million reads, using seqtk v1.3^1^ as previously described (Summers et al., 2019). Expression was then quantified as transcripts per million (TPM) using Kallisto v0.44.0 (Bray et al., 2016) with transcript-level expression estimates summed to the gene-level. To generate comprehensive Kallisto indices, we used (where available) the combined set of unique protein-coding transcripts from Ensembl and NCBI RefSeq as detailed in Supplementary Table S2.

For a meaningful cross-species comparison of expression levels, we also required a one-to-one relationship between gene names across species. This is complicated by the fact that some genes have multiple copies in one species but not others, as well as genomes differing in the completeness of the annotation. Should a gene name not be available in a given species, where possible we assigned a name on the basis of an ortholog in a near relative. For this purpose, orthology relationships were sourced from Ensembl BioMart (Kinsella et al., 2011) and required to be one-to-one, with ≥90% reciprocal identity and an ‘orthology confidence’ score of 1 (this score reflects a high whole genome alignment coverage and conservation of synteny, as described in Ensembl documentation^2^, accessed 30th March 2020). If there are multiple possible orthologs, Ensembl classifies the relationship between each member of the set as one-to-many. However, if only one member of a one-to-many set of genes met the other two criteria (of reciprocal identity and orthology confidence score), we reconsidered this gene to be the most probable one-to-one ortholog. Genes renamed on the basis of orthology are indicated in Supplementary Table S3. These automatically assigned orthology relationships were only made within two sub-groups of the closest related species in the dataset: three ruminants (sheep, cow, and goat), and the three rodents (*M. musculus*, *M. spretus*, rat). No orthologs were sought for horse or pig (being relatively distant species) or buffalo (not yet available via Ensembl). The final dataset includes 9,478 genes for which there are candidate orthologs in at least 8 of 9 species (Supplementary Table S3). Of these, 8,249 genes were annotated in all 9 species and expressed >10TPM in at least one sample. These are shown as a list ranked on maximal expression in Supplementary Table S4. Transcription factors within this comparative dataset were identified based on the curated list of 1,639 known or likely human transcription factors published by Lambert et al. (2018).

### Network and GO Term Enrichment Analysis

Network analysis was performed using Graphia, a computational tool which enables visualization and analysis of large correlation networks. This program is now available open-source as BioLayout.^3^ The data provided in Supplementary Tables can also be reanalyzed with a new version of Graphia.^4^ Networks created by Graphia were used in two ways. A sample-to-sample network was created to assess relationships between the nine species based on shared gene expression patterns between samples. The correlation co-efficient threshold of 0.8 was chosen to include all samples in the network. Gene-to-gene networks were created to determine co-expressed genes across all samples for the sheep BMDM time course and for the nine species responding to LPS. The correlation co-efficient for each of these gene co-expression networks (GCN) was chosen to optimise the number of nodes (transcripts) while minimizing the number of edges (correlations between nodes at or above the chosen correlation co-efficient) as shown in Supplementary Figures S1, S2. Enrichment of gene ontology (GO) terms for genes within the sheep clusters was assessed using DAVID,^5^ with *Ovis aries* as the background. Only enrichments with Benjamini–Hochberg adjusted *p*-value of ≤0.05 were considered significant.

## Results

### Response of Sheep Macrophages to LPS

**FIGURE 1:**
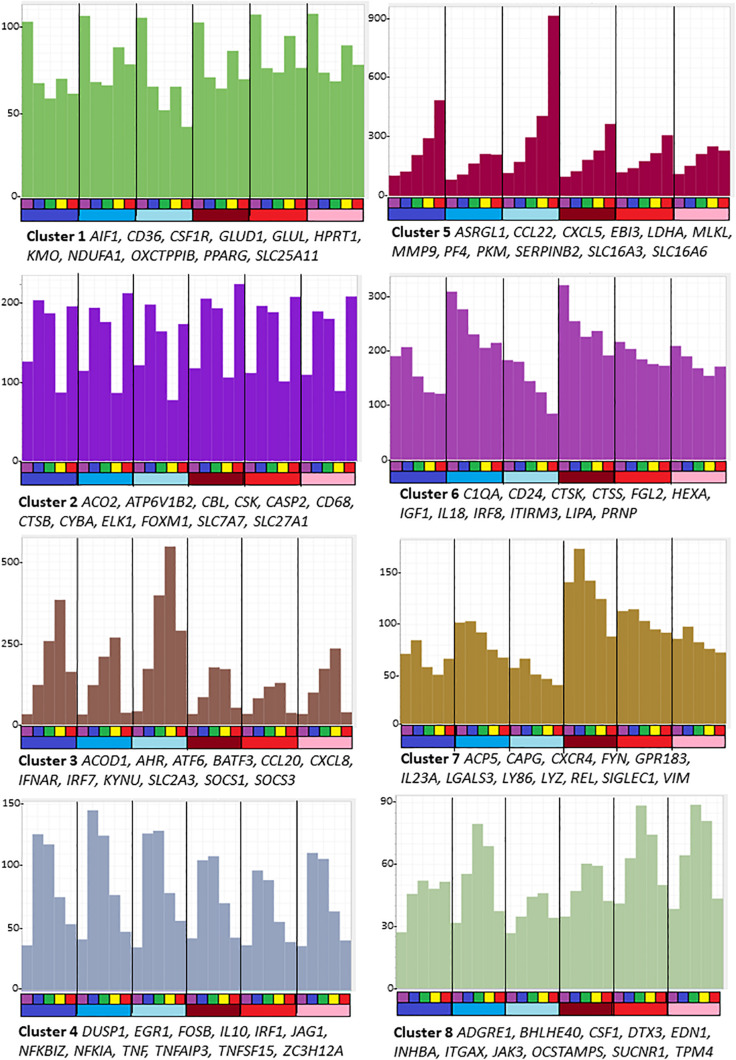
Average gene expression profiles and genes of interest within the clusters of the largest eight clusters (>80 nodes) from the analysis of sheep BMDM treated with LPS. The primary data are in Supplementary Table S5, and full list of clusters and co-expressed transcripts is provided in Supplementary Table S6. Graphia network analysis performed at a Pearson correlation threshold *r* ≥ 0.75 and MCL inflation 1.7. The *Y*-axis shows average expression (TPM) of genes in each cluster which indicates the shared pattern driving the correlated expression. For example, Cluster 5 contains transcripts that were each progressively up-regulated by LPS in all 6 animals. Genes named under each panel are representative of each cluster. The bars below the *X*-axis identify the samples. Each segment is the time course of an individual animal. The upper bar indicates the time point after adding LPS: purple = 0 h; blue – 2 h; green – 4 h; yellow – 7 h; red – 24 h. The lower bar indicates the sex of each individual: blue – males; red – females.

Graphia is a computational tool which enables the analysis and visualization of large correlation networks. Amongst many applications it was used to identify cell and tissue-specific co-expression clusters in the sheep (Clark et al., 2017), pig (Summers et al., 2019) goat (Muriuki et al., 2019), water buffalo (Young et al., 2019), and chicken (Bush et al., 2018a) transcriptional atlas projects. Co-expression analysis of a detailed time course of the transcriptomic response of human MDM to LPS treatment based upon sequencing of CAGE (genome scale 5′ RACE) libraries revealed the transient induction of positive and negative regulators (Baillie et al., 2017). For the time course of sheep BMDM responding to LPS treatment, we chose 5 time points, 0, 2, 4, 7, and 24 h, to cover the major peaks identified in the human study. The full dataset for the six animals is provided in Supplementary Table S5. The 6 animals varied to some extent in the degree and temporal profile of their response to LPS. This variation increased the power to identify transcript clusters that were strictly co-regulated. For the purpose of this analysis, only transcripts detected above 10 TPM in at least one library were included. The expression data were clustered at an optimal Pearson correlation co-efficient of 0.75, which includes 9,304 transcripts in the network. The lists of genes in each cluster and the average expression profiles are provided in Supplementary Table S6. Figure 1 shows the profiles for the eight largest clusters of interest annotated with specific index genes. With only 3 male and 3 female sheep in the dataset, it would not have been possible to detect subtle sex-specific gene expression but there was no obvious cluster that distinguished males and females.

Genes in the largest cluster, Cluster 1, containing more than half the total transcripts, were expressed constitutively and marginally down-regulated within 2 h by LPS. Cluster 6 and Cluster 7 each contain transcripts that were progressively down-regulated over the time course in all replicates, but the basal expression varied amongst the individual animals. These three clusters each contain multiple known macrophage-enriched transcripts (*CSF1R*, *C1QA*, *LGALS3*, and *IRF8*). Cluster 1 contains many components of the vacuolar ATPase, lysosomal enzymes and surface markers as well as the cell cycle transcriptional regulator *Foxm1* and many cell cycle-related transcripts (Giotti et al., 2019) and mitochondria-associated genes. Enriched GO terms and pathways include those related to lysosomes, endo- and exosomes, signaling, and cellular movement (Supplementary Table S6).

Cluster 2 contains transcripts that have a complex pattern of apparent transcriptional regulation; these were induced transiently at 2 h but then substantially and transiently down-regulated in all six replicates at 7 h.

Cluster 3 is the reciprocal to Cluster 2 and contains the largest set of inducible transcripts. The average expression of genes in this cluster peaks at 7 h and declines thereafter. It includes transcripts encoding transcriptional regulators, notably *AHR*, *ATF6*, *BATF3*, *EHF*, *IRF3*, *IRF7*, *IRF9*, and *STAT2* and numerous known interferon-inducible genes that are also induced by LPS in human macrophages, through autocrine IFNβ signaling and the MYD88-independent pathway (Baillie et al., 2017). The two type 1 interferon receptor genes (*IFNAR1/IFNAR2*) were highly expressed in unstimulated cells and the interferon-inducible feedback regulators, *SOCS1* and *SOCS3*, were induced by 2 h. However, the IFNβ genes in sheep are not currently annotated in Ensembl and the three IFNβ2-like transcripts did not meet the 10 TPM expression threshold at any time point. As such, the precise nature of the autocrine signal in sheep BMDM, if it exists, is unclear. Cluster 3 also contains transcripts encoding the classical pro-inflammatory cytokines *IL1B* and *IL6* and multiple chemokine genes (*CCL3*, *5*,*8*,*20; CXCL8*,*10*). The chemokine genes, notably *CXCL8* (encoding IL8), are amongst the most abundant transcripts in the LPS-induced state, collectively contributing > 100,000TPM. GO terms relating to antiviral response were enriched in this cluster.

Cluster 4 contains 395 transcripts that on average were induced transiently, peaking after 2–4 h and declining to basal levels by 24 h. These transcripts can be subdivided into several classes. They include classical early response genes encoding transcription factors (*EGR1*,*3*,*4; FOSB*, *FOSL1*, *FOSL2*, *IER3*, *IER5*, *JUNB*, *KDM6B*, *MYC*, and *NFKBIZ*) and negative regulators that were also induced in human MDM in response to LPS (*BCL3*, *BCOR*, *CISH*, *DUSP1*, *DUSP5*, *DUSP10*, *GADD45B*, *IL10*, *MEFV*, *NFKIA*, *NFKIB*, *SMAD7*, *SOC1*, *SOCS3*, *TNFAIP2*, *TNFAIP3*, *ZFP36*, and *ZC3H12A*) (Baillie et al., 2017) and were inferred to provide intrinsic limitation of the pro-inflammatory activation. The set of transient early response genes also included pro-inflammatory mediators such as *TNF* and *PTGS2*, and likely feed-forward regulators such as *IRF1.* A related cluster, Cluster 34, showed a similar average pattern but with more extreme induction from undetectable basal levels. It includes *CXCL1*, *JAG1*, *TNFSF15* and transcription factors *ETS2* and *KLF5.* More than 50 of the transcripts within Cluster 4 were unannotated on Ensembl. Around half of these could be confidently assigned gene names based upon clear orthology to human genes. Provisional annotations are shown in Supplementary Table S6. Amongst the most inducible and highest expressed were likely orthologs of transcription factors *CEBPD* and *KLF2.*

As in human macrophages responding to LPS, there was also a set of transcripts that continued to increase across the time course. Cluster 5 is the reciprocal cluster to Clusters 6 and 7. Transcripts in this cluster, as well as a subset of those in Cluster 3, are likely associated with resolution of inflammation. Amongst the most highly induced transcripts are those encoding matrix metalloproteinases, *MMP1*, *MMP3*, *MMP9*, *MMP12*, *MMP13*, *MMP14*, and *MMP25*. This cluster also contains ENSOARG00000006889, described as plasminogen activator inhibitor 2 and orthologous with *SERPINB2* of other mammals. *SERPINB2* is amongst the most highly induced transcripts in human monocytes (Baillie et al., 2017) and mouse macrophages (Costelloe et al., 1999) responding to LPS. There appears to be a gene duplication in ruminant genomes. Two *SERPINB2* family members have been found in cattle (ENSBTAG00000023198, described as serpin family B member 2, and ENSBTAG00000023026, described as serpin family B member 2-like). The sheep gene ENSOARG00000005159, described as plasminogen activator inhibitor 2-like, another sheep ortholog of *SERPINB2* in other mammals, also appears in Cluster 5 and was strongly induced by LPS.

As noted previously (Young et al., 2018) the sheep BMDM did not show induction of *NOS2* mRNA, nor of the inducible arginine transporter *SLC7A2* at any time point. Unlike human and pig macrophages, sheep BMDM expressed GTP cyclohydrolase (*GCH1*), required for the production of the NOS2 cofactor tetrahydrobiopterin, constitutively at low levels, but it was not LPS-inducible. In common with other ruminants (Young et al., 2018), sheep macrophages expressed the gene for the mitochondrial arginase enzyme, *ARG2*, which was substantially induced by LPS. *ARG2* is usually associated with the urea cycle in the liver and kidney (Caldwell et al., 2018). Annotation of the unannotated transcripts in Cluster 4 revealed that some are likely non-coding and have no orthologs in other species. Others reflect an issue also encountered in the recent pig atlas project (Summers et al., 2019) in which different Ensembl gene IDs are assigned to partial sequences/duplicates of known genes. For example, there are three *SLC7A1* transcripts in the sheep reference transcriptome, each showing the same pattern of rapid induction by LPS. *ENSOARG00000012028* maps immediately downstream of the annotated *SLC7A1* gene. Combining the TPM counts indicates that this transporter was, in fact, quite highly inducible by LPS. SLC7A1 transports arginine and ornithine. Ornithine decarboxylase (*ODC1*) was also highly expressed and further inducible in sheep macrophages. The high expression of ornithine amino transferase (*OAT*), which leads to the production of glutamate, suggests the main function of this pathway is to use arginine and ornithine as a fuel and support the unique arginine-urea biology of ruminants (Marini et al., 2004).

In humans and pigs, LPS promotes the uptake and metabolism of another amino acid, tryptophan, and its catabolism via the coordinated induction of three enzymes, indoleamine dioxygenase (IDO1), kynurenine monooxygenase (KMO) and kynureninase (KYNU) (Kapetanovic et al., 2012, 2013; Schroder et al., 2012). Curiously, *IDO1* mRNA was barely detected and was not LPS-inducible in sheep BMDM, whereas *KYNU* and *KMO* were highly expressed and *KYNU* was further induced by LPS. Kynurenine has assumed greater interest since the recognition of its role in immune modulation as an activator of the aryl hydrocarbon receptor (AHR) (Sinclair et al., 2018). *SLC7A5*, encoding a transporter which is required for the uptake of kynurenine, was highly inducible by LPS and peaked ahead of *KYNU*. Previous studies in other cell populations in sheep indicate that induction of *IDO1* depends upon stimulation with IFNγ (Entrican et al., 2009) which might synergise with LPS.

### Metabolic Regulation in Sheep Macrophages

The emerging field of immunometabolism has focused on the regulation of intermediary metabolism in recruited monocytes and macrophages in various states of activation or polarization. Amongst emerging concepts is the view that M1 polarization (classical activation) is associated with aerobic glycolysis and mitochondrial dysfunction, whereas M2 polarization requires an active TCA cycle (Ryan and O’Neill, 2020). This M1/M2 dichotomy is not well-supported by transcriptome analysis in mice and humans or in other species which instead favors a broad spectrum of activation states (Hume, 2015; Murray, 2017). CSF1 as the sole stimulus is sometimes considered an M2 agonist (Murray et al., 2014). An alternative view is that CSF1 drives a differentiated state that resembles the resident macrophages of the wall of the gut (Baillie et al., 2017). LPS on the other hand is classed as an M1 agonist but is usually considered in combination with the classical Th1 lymphokine, IFNγ (Murray et al., 2014; Murray, 2017). In any case, there is no support for the regulation of mitochondrial function in macrophages in the sheep transcriptomic data. Cluster 1 contained the large majority of mitochondria-associated transcripts (and was enriched for GO terms and pathways relating to mitochondrial function and metabolism; Supplementary Table S6). The mitochondria-associated transcripts were highly expressed and there was no evidence of major up or down-regulation by LPS.

In many cases metabolic pathways are regulated at the level of solute transport (Curi et al., 2017). There were 150 annotated members of the large solute carrier (SLC) family detected in the sheep BMDM, of which 71 were within Cluster 1 and mainly expressed constitutively. Macrophages in mice depend to varying degrees upon glutamine, glucose and fatty acids as fuels and glutamine is an important immune regulator (Liu et al., 2017). 14 different solute carriers from 4 families have been shown to transport glutamine (Bhutia and Ganapathy, 2016). Of the genes encoding these carriers, *SLC38A1*, contained within Cluster 3, was highly expressed and further inducible in the sheep BMDM. *SLC7A7* was constitutively expressed and *SLC1A5* and *SLC7A5* were both highly inducible within 2 h (Cluster 4). Consistent with the importance of glutamine as a fuel, transcripts encoding enzymes and mitochondrial carriers for glutamine metabolism (*GLS*, *GLUD1*, *GLUL*, and SLC25A11) were also highly expressed by sheep BMDM. One novel feature of the sheep BMDM was the very high expression of L-asparaginase (*ASRGL1* gene), which was further up-regulated later in the LPS response. Asparaginase may also possess glutaminase activity (Chan et al., 2014) and asparagine is likely taken up by SLC1A5 (also known as ASCT2). In humans, asparagine is a non-toxic carrier of residual ammonia to be eliminated from the body and regulates the uptake and metabolism of other amino acids, serine, arginine, and histidine, and thus protein and nucleotide synthesis (Krall et al., 2016). Interestingly, the *SDS* gene, encoding serine dehydratase, was also highly inducible by LPS. In non-ruminants, this enzyme is exclusive to the liver^6^ and deaminates serine to pyruvate and threonine to 2-ketobutyrate to provide substrates for gluconeogenesis.

The use of glucose as a fuel is regulated primarily at the level of glucose transport. In mice, a myeloid-specific conditional deletion of *Slc2a1* confirmed that the encoded protein GLUT1 is the major glucose transporter in macrophages but the loss of glucose as a fuel had remarkably little impact on macrophage function (Freemerman et al., 2019). In the sheep BMDM, *SLC2A1* was up-regulated by LPS, but another transporter *SLC2A3* (GLUT3) was more highly expressed and was induced further within 2 h. *SLC2A6* is a lysosome-associated glucose transporter that was recently knocked out in the mouse genome (Caruana et al., 2019). It was induced >10-fold by 2 h in response to LPS. The glycolytic activator 6-phosphofructose-2-kinase/fructose-2,6-bisphosphatase encoded by *PFKFB3* is proposed to upregulate glycolysis and link glucose metabolism to cell proliferation and survival in mouse macrophages (Jiang et al., 2016). *PFKFB3* was highly expressed in the sheep BMDM and induced further by LPS. Pyruvate kinase, muscle (*PKM*, also known as *PKM2*) and both transcripts of lactate dehydrogenase (*LDHA/LDHB*), as well as *SLC16A3* (which encodes the monocarboxylate carrier MCT4) and *SLC16A6* were further elevated later in the response (Cluster 5). The TLR-inducible expression of *SLC16A3* is shared with mice, and in that species is proposed to mediate the export of lactate from glycolysis as part of a positive feedback mechanism (Tan et al., 2015).

In the TCA cycle, citrate is initially converted to *cis*-aconitate by mitochondrial aconitase 2 (*ACO2*). In LPS-stimulated mouse macrophages, the TCA cycle is diverted through the induction of a novel enzyme, *cis*-aconitate decarboxylase 1, encoded by *ACOD1* (also known as immune responsive gene 1; *IRG1*), which catalyzes the conversion of *cis*-aconitate to *cis*-itaconate (Ryan and O’Neill, 2020). *ACO2* was robustly expressed across the sheep BMDM time course, but not specifically regulated in response to LPS. *ACOD1* was induced by 4 hrs in response to LPS in all individual sheep but level of expression remained low. In mice, the induction of *ACOD1* leads indirectly to the accumulation of the downstream TCA cycle intermediates succinate, fumarate and malate. Succinate may be an important metabolite in innate immune signaling which enhances IL1B production (Tannahill et al., 2013). Interestingly, the G-protein-coupled receptor for succinate from the TCA cycle (encoded by *SUCNR1*, also known as *GPR91*) was highly upregulated in LPS stimulated sheep BMDM, whereas it is undetectable in mouse BMDM.

Given the unique metabolism of ruminants, we considered the possibility that ketone bodies would be a preferred fuel for sheep macrophages. In mice, *Slc27a1*, encoding the fatty acid transporter FATP1 [which also contributes to functional regulation in macrophages (Johnson et al., 2016)] is highly expressed in BMDM alongside carnitine acyl transferase genes (*Crat*, *Crot*) and repressed by LPS. In sheep BMDM, *SLC27A1* and *SLC27A2* were just detectable, but *CD36*, which encodes a transporter for long chain fatty acids, was constitutively highly expressed. *SLC16A3* may also mediate uptake of the ketone body, acetoacetate, in exchange for lactate (Dimmer et al., 2000). *OXCT*, encoding succinyl CoA:3-oxoacid CoA transferase, which catalyzes the first step in ketolysis, was also expressed constitutively.

### Expression of Non-coding RNA in Sheep Macrophages

Genome-wide studies in multiple species have indicated that mammalian genomes are pervasively transcribed. Aside from protein-coding genes, several novel classes of non-coding RNAs (ncRNAs) contribute to transcriptional and translational regulation. Several thousand unique microRNAs have been identified in ruminant species (Bourdon et al., 2019). These have not been captured in our pipeline unless we captured their precursors. Another class of non-coding RNAs is derived from the transcriptional activation of enhancers; these were identified in LPS-stimulated mouse macrophages (Kaikkonen et al., 2013) and were also identified in genome-scale 5′RACE (CAGE) in human monocyte-derived macrophages responding to LPS (Baillie et al., 2017). These transcripts are rapidly degraded from the 3′ end by the exosome complex and are generally detected at <10 TPM with CAGE, and much lower with total RNA-seq. The final class of transcripts of interest is the long intergenic non-coding RNA (lincRNA). These have been attributed roles in transcriptional regulation and chromatin structure [reviewed in Ransohoff et al. (2018)]). By contrast to protein-coding mRNAs, they are commonly expressed at low levels but are more tissue or cell-type restricted. We recently combined RNA-seq data from multiple ruminant species to identify a consensus set of 5,350 lincRNA (Bush et al., 2018b). Of these predicted lincRNA, >4000 were detected in sheep BMDM, but the majority of these were expressed at <1 TPM (Supplementary Table S7). Only 230 were expressed >10 TPM in either the stimulated or unstimulated states; these are generally annotated as ‘RNA gene’ in Ensembl. Of the lincRNA, 54 were up-regulated > 2-fold and 13 down-regulated > 2-fold with LPS. We did not detect any obvious co-localisation of any inducible lincRNA with inducible protein-coding transcripts. Although mature microRNA were not detected because of the mRNA isolation protocol used, one of the most inducible non-coding RNA transcripts (MSTRG.36731) overlaps the large microRNA cluster on sheep chromosome 18 (18:64641285–64642220) suggesting that these microRNA have a role in innate immune regulation.

### Comparative Analysis of Macrophage Gene Expression

Previous comparative analysis of inducible gene expression in BMDM from multiple species confirmed major differences in regulated expression of genes involved in arginine metabolism (Young et al., 2018). The large majority of analysis of transcriptional regulation and gene function in macrophages has been carried out in mouse and to a lesser extent in humans. To extend the cross-species comparison, we downloaded expression data for BMDM with and without TLR4 stimulation from 8 species in addition to sheep, and requantified expression as described in Section “Materials and Methods.” The genome of each species has been annotated to a different extent and, as discussed for the sheep above, in many cases likely orthologies have not yet been adopted as gene names in Ensembl.

Supplementary Table S3 contains the full list of 10,770 genes expressed in at least one species for which we were able to identify orthologs in at least 3 species. The chosen time point to analyze the LPS response across mammalian species was 7 h in the large animals and rat and 6 h in the two mouse species. This captures the peak of response in the sheep where the majority of induced genes were increased in all 6 animals, consistent also with data from the human MDM time course (Baillie et al., 2017). This choice does omit analysis of immediate early genes (e.g., some genes within Clusters 4 and 34 in the sheep time course) where the induced expression had declined to baseline by 7 h.

There are averaged data for at least 3 animals in each species dataset and the culture conditions were almost identical for all species other than mouse. However, we know from previous studies in the pig (Kapetanovic et al., 2013), mouse (Raza et al., 2014; Buscher et al., 2017), and human (Fairfax et al., 2014) and the present study in sheep that there is considerable variation in the level of transcript expression and temporal profile between individuals and strains. As a first approximation to identify conserved regulation, Supplementary Table S3 ranks the average fold induction of each gene for each species and then summarizes the sum of ranks, the range of ranks and describes the pattern of species-specificity. A low sum of ranks provides an indication of consistency of induction across species, and the top 10 genes induced by LPS on that basis are *IL1B*, *CXCL10*, *IL6*, *CCL5*, *ISG15*, *RSAD2*, *ACOD1*, *IL1A*, *IFIT1*, and *IL27* while the highest ranked transcription factor genes are *IRF1* and *IRF7.* Even within that set of most inducible transcripts the level of expression was highly variable amongst species. Notably, *IL1B* was very lowly expressed in pig, and *CXCL10*, *CCL5*, *RSAD2*, *ACOD1*, and *IRF7* were lowly expressed in sheep and goat. We were able to identify 8,240 genes for which there was a likely ortholog in all 9 species and in which expression was >10 TPM in at least one sample. These are provided separately in Supplementary Table S4 ranked in order of maximum level of expression in any sample.

To determine whether the transcriptional basis of macrophage differentiation and activation was conserved, the profiles of genes encoding transcription factors (TF) were analyzed separately. A total of 421 TF genes were expressed in all 9 species at >10 TPM in at least one sample (Supplementary Table S8). The molecular basis of gene regulation during macrophage differentiation in mouse has been reviewed extensively (Fonseca et al., 2016; Hume et al., 2016; Monticelli and Natoli, 2017; Rojo et al., 2017). Although there were minor differences in the level of expression between the species, the core TF network was well-conserved. Master regulators such as *SPI1* (encoding the lineage-specific TF PU.1) and *MAFB* were constitutively expressed in BMDM in all species. The three members of the MITF family (*TFEB*, *TFEC*, and *MITF*), all of which are expressed in mouse macrophages (Rehli et al., 1999) and bind to regulatory elements in the promoters of many lysosome-associated transcripts (Hume et al., 2010) were also expressed in all species. One core TF absent from the conserved list was *CEBPB.* Annotation for this factor is missing in sheep. A BLAST search on Ensembl using the cow gene identified *ENSOARG00000013395* as the likely ortholog in sheep and as expected it was constitutively expressed in sheep BMDM and further induced by LPS as in all 8 other species (Supplementary Table S3). We conclude that the transcriptional network that controls macrophage differentiation and LPS responsiveness is largely conserved across mammals. In the light of discussion above about the role of interferon in sheep BMDM, one important exception is *IRF7.* In human monocytes, quantitative variation in LPS-inducible transcripts is associated with the level of *IRF7* (Hume and Freeman, 2014). As noted above, sheep and goat BMDM expressed very low levels of *IRF7* whereas it was highly inducible in all other species.

**FIGURE 2:**
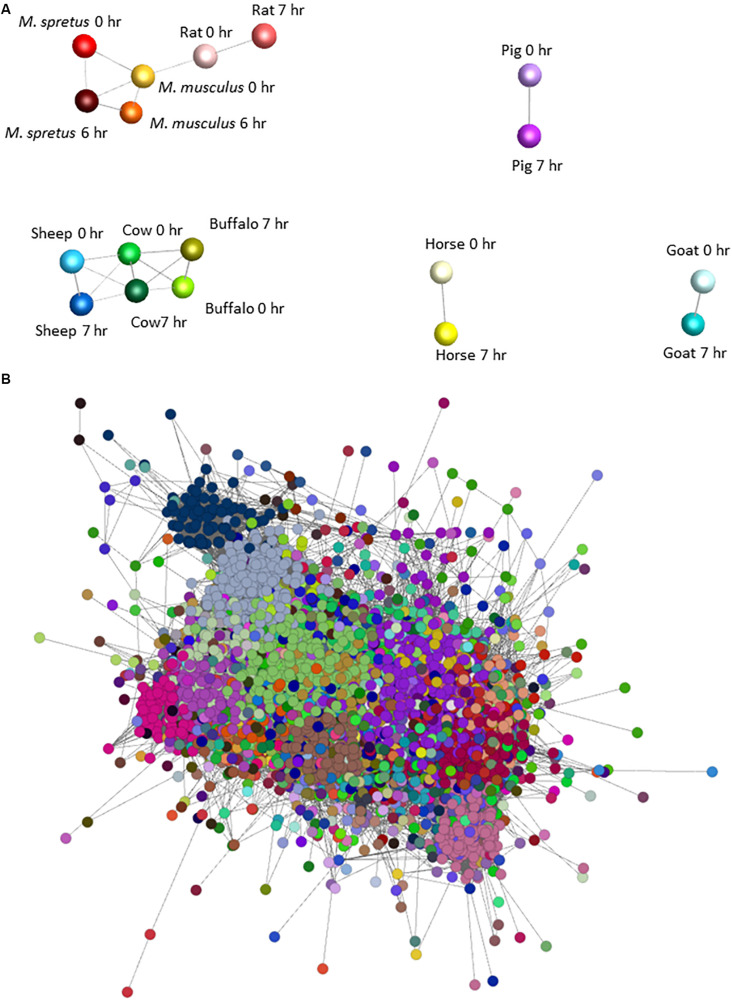
Network analysis of BMDM from nine mammalian responding to stimulation with LPS. **(A)** Sample-to-sample 3D network of the BMDM treated with LPS. The Pearson correlation threshold was *r* = 0.8. Each node represents the BMDM from a species at 0 and 6 or 7 h post LPS exposure and the lines between them are connections above the threshold correlation coefficient. The layout demonstrates the separation of pig, goat and horse, and the close relationship among rodent samples and ruminant samples, and between the control and LPS-stimulated samples of each species. **(B)** Network graph for gene coexpression network for BMDM from each species with and without LPS stimulation. The Pearson correlation threshold was *r* = 0.8, MCL inflation value 1.7. Each node is a gene and the lines between them are connections above the threshold correlation coefficient. Nodes (genes) highlighted with the same color represent co-expression clusters determined by the MCL clustering algorithm with an inflation value of 1.7. Note that this is a 2D representation of a 3D network graph. The average expression profiles of genes within the largest clusters are shown in Figure 3.

**FIGURE 3:**
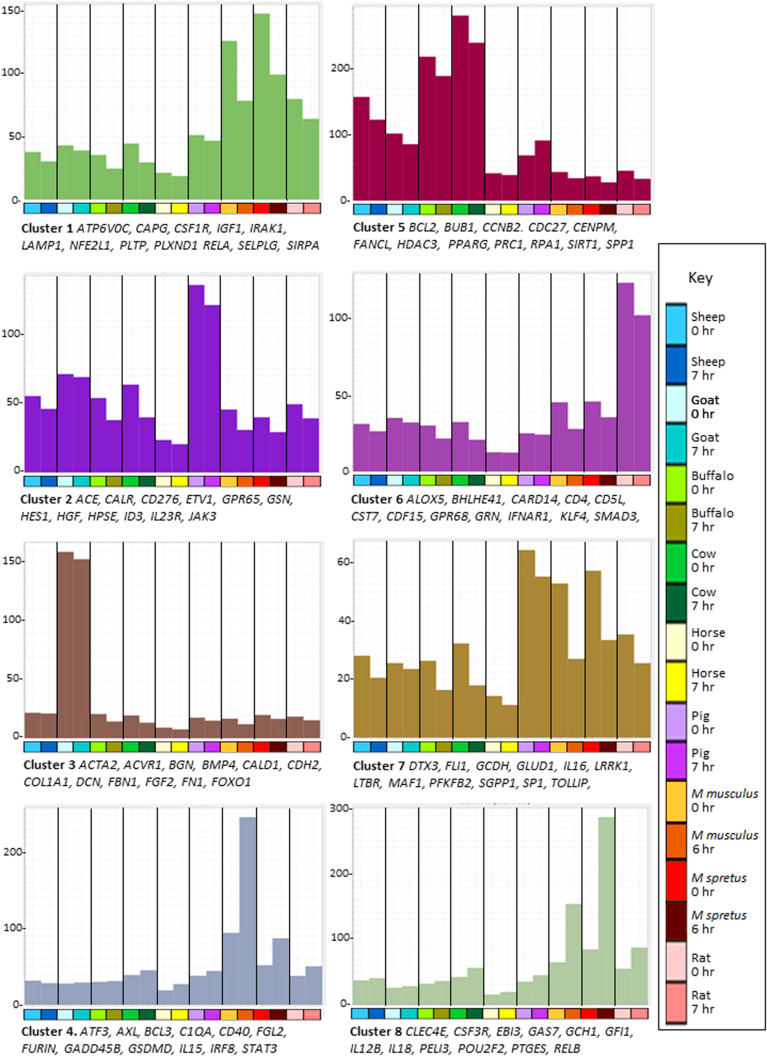
Network analysis of BMDM from nine mammalian species responding to stimulation with LPS. Average gene expression profiles and genes of interest within the clusters of the largest eight clusters (>170 nodes) from the gene-to-gene analysis of BMDM with or without LPS shown in Figure 2B. Graphia network analysis performed at R ≥ 0.8, MCL inflation 1.7. *Y*-axis shows average expression of genes in each cluster (TPM) which indicates the shared pattern driving the correlated expression. For example, the basal expression of transcripts in Cluster 1 was enriched in the rodent BMDM and was down-regulated in all species by LPS. Genes named under each panel are representative of each cluster. The full set of genes in each cluster and all smaller clusters is provided in Supplementary Table S9. *X*-axis shows the samples. Bar indicates species: dark blue – sheep; light blue – goat; light green – buffalo; dark green – cow; yellow – horse; purple – pig; red – *Mus musculus*; dark red – *Mus spretus*; pink – rat. For each species the first column shows the average gene expression at 0 h and the second column shows the average gene expression after LPS treatment; 6 h for *M. musculus* and *M. spretus*; 7 h for all other species. Colors of the bars in the graphs are the same as those of the genes in that cluster in Figure 2B.

To identify sets of transcripts that vary in parallel between species we again used Graphia. Figure 2A shows the sample-to-sample matrix for the complete dataset. The graph reveals that control and LPS-stimulated datasets for three of the ruminant samples (sheep, cow, and water buffalo) cluster together as do the three rodent datasets. The goat dataset clustered separately from the other ruminants for reasons that become evident below, and pig and horse were also distinct. Figure 2B shows the GCN and Supplementary Table S9 the contents of each of the clusters and their average expression profiles. Profiles of the largest clusters and index genes in each are shown in Figure 3. The two largest clusters contain genes that on average are constitutively expressed and marginally down-regulated by LPS. Cluster 1 was enriched around twofold in the rodent macrophages. It contains the macrophage-specific growth factor receptor, *CSF1R.* Cluster 2 and Cluster 7 have a similar profile to Cluster 1 but genes within them were on average more highly expressed in the pig BMDM relative to the ruminants and horse. Genes in Cluster 3 were highly expressed only in the goat BMDM and the cluster was strongly enriched for collagens and other mesenchyme-associated transcripts. These goat BMDM cultures were generated from 6 days old animals (Supplementary Table S1) when the marrow hematopoietic compartment may not have been fully established with residual mesenchymal cells contributing to the RNA pool. Cluster 5 is clearly enriched with genes previously annotated as cell cycle related (Giotti et al., 2019). The average expression was higher in the BMDM cultured from the four ruminant species and pigs and presumably reflects an ongoing proliferation at the time of harvest. Most transcripts in the cluster were repressed by LPS, consistent with the known growth inhibitory impacts of TLR signaling (Sester et al., 2005).

The average expression profiles of many of the smaller clusters vary between species, either in basal expression or pattern of regulation. Even within species, individual loci exhibit heritable differences in the level of expression (Fairfax et al., 2014). Species-enriched clusters such as Cluster 10 (buffalo), Cluster 11 (sheep), Cluster 12 (rat), Cluster 14 (cow), Cluster 16 (horse), and Cluster 36 (pig and horse) do not contain obvious transcriptional regulators that might act in *trans* so most species-specific variation is likely *cis-*acting. Supplementary Table S9 shows the profiles and gene lists for these clusters.

Previous studies have indicated the substantive differences in regulated gene expression in macrophages between mice and large animals (humans and pigs) (Kapetanovic et al., 2012; Schroder et al., 2012). Clusters 4, 6, and 8 further highlight the difference between the rodent and large animal BMDM. Cluster 4 was enriched in the rodent BMDM, especially *M. musculus*, and further-induced by LPS. It includes TF genes *ATF3*, *CREB5*, *ETV6*, *FOXP1*, *IRF8*, and *STAT3.* Some transcripts within Cluster 4 (e.g., *C1QA* and *C1QB*) reflect the idiosyncrasies of the C57BL/6 mouse strain in which many transcripts are over-expressed or absent compared to other strains (Raza et al., 2014). Transcripts within Cluster 6 were highest in rat BMDM, again including multiple transcriptional regulators: *BHLHE41*, *CREB3*, *CUX1*, *ESRRA*, *ETV3*, *KLF4*, *NFATC1*, and *SMAD3.* Transcripts within Cluster 8 were also, on average, more highly expressed in rodents, and highly inducible by LPS. This cluster contains the rodent-specific macrophage marker *ADGRE1* (F4/80) and includes several lineage-enriched TF genes such as *AKNA*, *GFI1*, *KLF3*, *LMO4, NR1H3* and *SPI1.*

Several smaller clusters contain the transcripts that on average were induced by LPS in all species but with differences in relative activation. Clusters 37 and 39 contain most of the highest-ranked inducible transcripts in Supplementary Table S3, including *IL1A* and *IL1B.* Cluster 9 contains transcripts induced in all species but most highly in bovids (cow and water buffalo), including *BATF3*, *JUN*, *HIF1A*, *MITF*, *NFE2L3*, *NFIL3*, *PRDM1*, *STAT1* and transcripts encoding inflammatory effectors *CSF3* and *PTX3.* Transcripts in Clusters 19 (containing *IRF1*, *NR3C1*, and *REL*) and 38 (containing *IRF7*) were induced in all species but considerably less so in sheep and goats. This suggests that the comparatively low LPS-induced expression of a subset of interferon target genes discussed above is related to low expression of the key TFs IRF1 and IRF7. Similar variation in IRF gene family expression between individual humans (Hume and Freeman, 2014) and chickens (Freem et al., 2019) has also been associated with co-regulated expression of IRF target genes. The reciprocal Cluster 25 contains transcripts induced specifically in sheep and goat BMDM. It does not include obvious *trans*-acting factors, suggesting that these differences are locus-specific.

## Discussion

### Feedback Regulation of the LPS Response in the Sheep

The analysis of the response of sheep BMDM to LPS confirms a pattern that was evident from a more extensive analysis of the response of human MDM to LPS (Baillie et al., 2017). The LPS response is a temporal cascade in which each peak of regulated gene expression includes both feed-forward activators and feedback inhibitors. The sheep BMDM response to LPS shares with the human macrophage response the rapid induction of numerous immediate early transcription factors. Amongst the most-inducible was the atypical NF-κB inhibitor protein gene, *NFKBIZ*, the product of which (IκBζ) interacts with Akirin2 to *trans*-activate inflammatory cytokine genes such as *IL6* and *IL12B* (Tartey et al., 2014). The canonical MyD88-independent TRIF/TRAM (TICAM1/2)-dependent pathway of IFN regulation determined from studies of mice involves the interactions of TRAF3 and the kinase TBK1 to phosphorylate IRF3, but this pathway is not conserved in humans (Schroder et al., 2012). In the sheep BMDM also, *IRF3*, *TBK1*, and *TRAF3* were expressed at very low levels and the most inducible member of the IRF family was *IRF1. TICAM1* was amongst the early LPS-responsive genes and in common with human, LPS induced *TRAF1.*The other feature shared with human macrophages was the rapid induction of genes encoding repressors of the TLR4 signal at multiple levels, including *BCL3*, *CISH*, *DUSP1/2/5/6*, *GADD45B*, *IL10*, *NFKB1/2*, *NFKBIA/B/D/E*, *PPP1R15A*, *SOCS1/3*, *TNFAIP3/6*, *TRIM25*, *ZFP36*, and *ZC3H12A.* Each of these inducible feedback regulators is shared with LPS-stimulated human MDM (Baillie et al., 2017). On the other hand, many of the negative regulators of TLR4 signaling identified in mice and discussed by Kondo et al. (2012) [e.g., *AHR*, *ATF3*, *ATG16L1*, *KLF3*, *MSK1/2*, *PDLIM2*, *PIN1*, *RPS6K* family (also known as MSP1/2)] were expressed constitutively in sheep BMDM as in human MDM (Baillie et al., 2017) and were not further-regulated by LPS. As discussed previously, the surprising feature of the feedback regulation of the LPS response is the apparent lack of redundancy, in that mutation or deletion of any of these regulators can lead to excessive TLR signals and inflammatory pathology in humans and model organisms (Wells et al., 2005).

### Conserved Transcription Regulation in Macrophages

A comparative analysis of gene expression in the liver of 25 mammalian species concluded that the transcriptional profile and spectrum of lineage-specific TFs is largely conserved (Berthelot et al., 2018) despite the gain and loss of individual regulatory elements. We hypothesized that cells of the innate immune system could display greater diversity between species than hepatocytes as a consequence of the evolutionary pressure of pathogen selection which would differ between species. Analysis of the response of mouse, human and pig macrophages to LPS (Schroder et al., 2012) and to glucocorticoids (GC) (Jubb et al., 2016) revealed the extensive gain and loss of *cis*-acting regulatory elements between the species that accounts for differences in inducible gene expression. This was especially obvious in the case of GC, where transcriptional activation derives from a single regulatory motif, unlike LPS-inducible gene expression which samples a smorgasbord of inducible TFs. The comparative analysis of the mammalian macrophage transcriptome presented here supports the evolutionary conservation of the overlying transcriptional network that drives macrophage biology. The set of constitutive and LPS-inducible TF transcripts was largely conserved across the 9 species we examined, and consistent with previous analysis of human MDM (Baillie et al., 2017).

As expected, amongst the most abundant transcripts shared by all macrophages are those encoding components of the endosome/lysosome pathway (e.g., *CD68*, *GPNMB*, *LAMP1*, and *NPC2*) and lysosomal hydrolases (*CTSB*, *CTSD*, *LGMN*, and *LIPA*). The largest divergence between the species separated the rodents from all the large animals and includes transcripts in Clusters 4, 6, and 8 (Figure 3). These differences need to be interpreted with some caution, because the clusters include multiple lineage-specific transcription factors such as *CEBPB*, *IRF8*, *NR1H3*, and *SPI1* that appear somewhat over-expressed in rodents, especially in the mouse samples. Unlike all of the other samples (which were generated by our group), the mouse BMDM were grown in L929 conditioned medium (as a source of CSF1) rather than recombinant CSF1, and in DMEM/high glucose with 20% fetal bovine serum (FBS) (Link et al., 2018) rather than RPMI 1640/10% FCS which is optimal for mouse BMDM proliferation (Hume and Gordon, 1983). One possibility we cannot eliminate is that the mouse BMDM are simply more fully differentiated as a result of the different culture conditions. Equally, there may be mouse-specific differences in CSF1 responsiveness, associated with the unique lack of autocrine *Csf1* expression by mouse BMDM (see above) which is also evident in independent datasets from our group.^6^ The reciprocal immature phenotype is reflected in the cluster of cell cycle-associated transcripts which remain more highly expressed in the macrophages cultured from the large animals (Cluster 5, Figure 3).

### Divergent Metabolic Regulation Amongst Mammalian Species

The shared high expression of genes involved in glutamine metabolism (*GLS*, *GLUL*, *GLUD1*, and *SLC25A11*) in BMDM from all species suggests that the use of this amino acid as a fuel by macrophages, demonstrated previously in mouse and human (Curi et al., 2017), is conserved amongst mammalian species. *SLC1A5*, which encodes a plasma membrane glutamine transporter, was also highly expressed in all the macrophage populations, especially ruminants (sheep and cow) where it was further induced by LPS. A variant isoform of SLC1A5 was shown recently to be involved in mitochondrial glutamine uptake in human tumor cells (Yoo et al., 2020).

Other metabolic pathways appeared more divergent. Our previous study documented the major differences in arginine metabolism and production of nitric oxide by these species and the underlying transcriptional basis for the difference (Young et al., 2018). Cluster 12 was most highly induced in the rodent BMDM and included *NOS2*, *ARG1* (encoding arginase 1), the gene for the inducible arginine transporter, SLC7A2, required for nitric oxide production in mouse macrophages (Kakuda et al., 1999) and arginosuccinate synthase (*ASS1*) which recycles citrulline to regenerate arginine (Qualls et al., 2012). As with *NOS2*, most of the genes in this cluster were also induced to a lesser extent in all the bovid BMDM in response to LPS and a temporal difference in the kinetics of induction cannot be eliminated. Interestingly, although *ACOD1* did not form part of Cluster 12, the level of induction by LPS was >10-fold lower in those species (goat, horse, pig, and sheep) in which *NOS2* induction was minimal. The induction of *ACOD1* may actually be a direct response to mitochondrial toxicity of NO (Jamal Uddin et al., 2016). The product of *ACOD1*, itaconate, induces *HMOX1* (Mills et al., 2018) which was also most inducible by LPS in the rat BMDM.

Notwithstanding the differential regulation of *IDO1* in sheep and goats, other genes involved in tryptophan metabolism (*KYNU*, *KMO*) were expressed and LPS-responsive only in BMDM from large animals, as shown previously in humans and pigs (Schroder et al., 2012). Accordingly, application of IDO1 inhibitors as therapeutic agents based upon rodent disease models (Prendergast et al., 2017) needs to be considered with caution. Kynurenine is an immune modulator, and studies in mouse T cells identified SLC7A5 as a transporter facilitating the uptake of this metabolite (Sinclair et al., 2018). In common with human macrophages where kynurenine has been attributed an anti-inflammatory role (Rotoli et al., 2018) an alternative kynurenine transporter, *SLC7A7* was constitutively expressed in BMDM from all the large animals. The macrophages from all species expressed high levels of the *SLC7A7* dimerization partner, *SLC3A2*, which forms the large/neutral amino acid transporter (y^+^ LAT1) complex.

We speculated *a priori* that ruminant macrophages might be adapted to use fatty acids or ketone bodies as fuels because of the high levels of these metabolites produced by ruminant fermentation. This profile might potentially be shared with horses, which produce volatile fatty acids from hind gut fermentation. There was no evidence to support this hypothesis. Any such adaptation may be obscured by use of a common cell culture medium containing high glucose and relatively high oxygen. BMDM from the four ruminant species did share regulated high expression of other metabolism-associated transcripts noted from analysis of the sheep BMDM time course, including *ARG2*, *ASRGL1*, *LDHB*, *SDS*, and *SUCNR1*. The LPS-inducible expression of serine dehydratase (SDS) that was unique to the ruminants may also contribute pyruvate to support oxidative metabolism. Unlike rodents, BMDM from all of the large animals shared expression of *SLC2A3* (encoding GLUT3) although the level of expression and response to LPS differed. In humans, *SLC2A3* was expressed in blood monocytes and down-regulated during their maturation to the CD16^++^ subset alongside increased oxidative metabolism (Schmidl et al., 2014). This pattern was recently confirmed in bovine monocytes (Eger et al., 2016). GLUT3 was first identified as a neuron-specific glucose transporter but subsequent studies revealed expression by human leukocytes and inducible translocation to the plasma membrane in response to stimulation [reviewed in Eger et al. (2016)]. GLUT3 has both the lowest *K*_m_ and the highest *V*_max_ of any of the glucose carriers, but it is unclear why this would be important for large animals compared to rodents. BMDM from all the species except horse also expressed the leukocyte-enriched hexokinase gene, *HK3.*

### Species Specific Immune-Related Genes

The merged data in Supplementary Tables S3, S4 and the cluster analysis of those (Supplementary Table S9) provide a resource to identify macrophage-expressed and LPS-inducible genes that are shared across all mammalian species. Other gene-specific differences amongst species may aid especially in the interpretation of studies using rodents as models to understand gene function. Table 1 provides examples of transcripts for which there is some evidence of innate immune function in rodents, and which were up-regulated in all the mammalian macrophages. They include many additional examples of inducible feedback regulators highlighted in analysis of the sheep time course. In Table 2, we identify examples of transcripts that were expressed and/or regulated in macrophages and clearly highly divergent between rodents and large animals. Some families of genes are discussed in more detail below.

**TABLE 1:** Summary of novel genes induced by LPS in all mammalian BMDM.

Gene symbol	Gene name/description	Gene function	References
ALCAM	Activated leukocyte adhesion molecule	Regulator of cell trafficking, interacts with CD6	Zimmerman et al., 2006; Renard et al., 2020
ADAR	Adenosine deaminase, RNA-specific	A to I editing of mRNA.	Baal et al., 2019
CASP7	Caspase 7	Regulator of NF-κB-dependent transcription	Erener et al., 2012
CD274	Programmed cell death ligand 1	Regulation of T cell tolerance	Yamazaki et al., 2002
CFLAR	CASP8 and FADD-like apoptosis regulator (aka c-FLIP)	Regulator of inflammasome	Van Opdenbosch et al., 2017; Muendlein et al., 2020
DTX3L	Deltex E3 ubiquitin ligase	Forms complex with PARP9, regulates IFN response	Zhang et al., 2015
EPSTI1	Epithelial stromal interaction 1	Unknown function. Negative regulator of macrophage activation	Kim et al., 2018
IL27/EBI3	Interleukin 27	IL12-related. Heterodimeric cytokine, feedback regulator of IFN response	Bosmann and Ward, 2013; Aparicio-Siegmund and Garbers, 2015
PDE4B	Phosphodiesterase 4B	Feedback regulator of LPS response	Jin et al., 2005
PML	Promyelocytic leukemia	Regulator of apoptosis Required for LPS response	Lunardi et al., 2011
PNPT1	Polyribonucleotide nucleotidyltransferase 1	3′–5′ exonuclease associated with mitochondria	Sarkar and Fisher, 2006; Wang et al., 2010
RNF114	Ring-type zinc finger 114	Negative regulator of NF-κB. Interacts with TNFAIP3	Rodriguez et al., 2014
RNF19B	Ring-type zinc finger 19B (aka NKLAM)	E3 ubiquitin ligase, associated with phagosomes	Lawrence and Kornbluth, 2012; Lawrence et al., 2019
SDC4	Syndecan 4	Transmembrane heparan sulfate proteoglycan, regulator of LPS response *in vivo*	Ishiguro et al., 2001
TDRD7	Tudor domain containing 7	Inhibitor of AMPK and autophagy	Subramanian et al., 2018
TRIM21	Tripartite motif 21	E3 ubiquitin ligase, Inhibitor of IRF transcription factors	Labzin et al., 2019; Sjostrand et al., 2020
TRIM 23	Tripartite motif 23	E3 ubiquitin ligase, regulator of autophagy	Sparrer et al., 2017

*Genes chosen were induced in all species and selected based upon limited related literature. References are primarily to studies in mice and provide evidence for a function in the response to LPS in vitro or in vivo.*

**TABLE 2:** Genes that show divergent regulation between large animals and rodents.

Gene symbol	Gene description	Gene function/regulation	Specificity	References
ADGRE1	Adhesion G protein- coupled receptor E1	F4/80 in mouse. Highly divergent expression, structure, sequence and function amongst species	Rodent	Waddell et al., 2018
CD163	Haptoglobin receptor	High in all large animals, absent from rodent BMDM. Regarded as M2 polarization marker.	Large animals	Vogel et al., 2014
CLEC4E	C type lectin 4E	Macrophage-induced C type lectin (Mincle). Role in fungal resistance induction by LPS is rodent-specific. Clec4 family expanded in rodents.	Rodent	Wells et al., 2008
C1QA, C1QB, C1QC	Complement 1Q	Very high expression in *Mus musculus* BMDM, known C57BL/6 strain-dependent	C57BL/6 *Mus musculus*	Raza et al., 2014
CPM	Carboxypeptidase M	Phosphoinositol-linked ectopeptidase. Marker of human MDM maturation from monocytes. Not expressed in rodent BMDM.	Not in rodents	Rehli et al., 2000
CTSK	Cathepsin K	Key gene in bone resorption, very high in all BMDM except *Mus musculus* (C57BL/6)	Not in *Mus musculus*	Drake et al., 2017
ENPP1	Ectonucleotide pyrophosphatase/phosphodiesterase 1	Not expressed in rodent BMDM. Involved in regulation of calcification and signaling	Not in rodents	Roberts et al., 2019
MARCO	Macrophage receptor collagenous structure	Scavenger receptor. Mouse-specific, induced by LPS.	Mouse	Jing et al., 2013
MMP9	Matrix metalloproteinase 9	Inducible matrix degrading enzyme. Constitutive in all BMDM except mouse.	Not in mouse	Min et al., 2002
POU2F2	Oct-2 transcription factor	Expressed and induced by LPS only in rodent. Implicated in NOS2 regulation	Rodent	Lu et al., 2009
PTX3	Pentraxin 3	Humoral pattern recognition molecule. Many roles in inflammation. Not inducible in rodents.	Not inducible in rodents	Garlanda et al., 2018
SCIN	Scinderin (adseverin)	Gelsolin-related actin binding protein, implicated in osteoclast fusion in mice. High in all BMDM except rodents	Not in rodents	Wang et al., 2018
TNFAIP6	TNF alpha induced protein 6 (aka TSG6)	Secreted cytokine with IL4-like activity. Implicated in anti-inflammatory activity. Not induced by LPS in rodent BMDM	Not inducible in rodents	Mittal et al., 2016; Nepal et al., 2019
VSIG4	V set and immunoglobulin domain containing 4	Complement receptor (CRIg). Tissue resident macrophage marker. Low expressed in mouse BMDM	Low in mouse	Irvine et al., 2016

*Genes shown are expressed or regulated only in rodents or large animals. References are to functional studies of the gene product or reviews where available.*

One gene and potential function that is clearly divergent between rodents and large animals is *APOBEC1* which is involved in RNA editing of cytosine to uracil (Harjanto et al., 2016). This transcript was expressed constitutively only in mouse BMDM. In human MDM, *APOBEC1* was not expressed but *APOBEC3A* was highly LPS-inducible (Baillie et al., 2017) and has been shown to mediate RNA editing in human monocytes and macrophages (Sharma et al., 2015). The APOBECs are a multigene family and cross-species orthologies are difficult to establish. The putative *APOBEC3A* gene in sheep (annotated as *APOBEC3Z1*) was highly induced in only 3 of the sheep BMDM analyzed. The highly LPS-inducible cytidine deaminase (*CDA*) gene might fulfill this function in ruminant macrophages.

The set of around 2,500 genes for which we detected expression but could not identify orthologs in all species includes genuine copy number variants (some of which are discussed below) and inconsistent gene names that require manual curation. There is a subset annotated only in the large animals or in rodents (e.g., the zinc finger proteins) where ortholog relationships are ambiguous. The Fc receptor family (FCGR), which are clearly important for macrophage effector functions (Bruhns and Jonsson, 2015) have copy number variants that are functionally important in humans, with consequent confusion in orthology relationship with other species. There are many other genes currently annotated only as open-reading frames (Orf) in humans. The merged data confirms expression of these transcripts in macrophages of large animals. The large majority of these transcripts show no evidence of regulation in any species, likely reflecting the long-term focus of annotation efforts in all species on regulated genes. One exception is *C15orf48*, which was induced by LPS in all the large animal species and was also induced by LPS in human MDM (Baillie et al., 2017). Although the 83 amino acid Orf is conserved across species, this is also the host gene for miR-147, a microRNA induced by LPS in mouse macrophages and implicated in feedback regulation (Liu et al., 2009). *CXorf21*, expressed and further inducible by LPS in all the large animals, as in humans (Baillie et al., 2017), contains risk alleles for systemic lupus erythematosus and Sjögren’s syndrome (Harris et al., 2019; Odhams et al., 2019). Although not in the pseudoautosomal region, the gene escapes X inactivation and is proposed to underly increased female autoimmune disease prevalence in humans. The protein product may regulate lysosome acidification (Harris et al., 2019). Another gene lacking functional annotation is *C9orf72*, the major locus implicated in human amyotrophic lateral sclerosis. This gene was highly expressed in human MDM (Baillie et al., 2017) and a mouse knockout led to macrophage dysfunction and neuroinflammation (O’Rourke et al., 2016). *C9orf72* was expressed in BMDM from all the species.

In view of the central role of CSF1 in macrophage homeostasis, the gene encoding this growth factor is of particular interest. Mouse BMDM express a very low level of *Csf1* mRNA and depend upon exogenous CSF1 for survival (Hume and Gordon, 1983). In humans, *CSF1* mRNA is undetectable in monocytes but rapidly induced *in vitro* as they differentiate to macrophages (Baillie et al., 2017). In mouse BMDM cultured by our group and analyzed using arrays,^6^ *Csf1* mRNA was undetectable in untreated cells, but induced at 7 h by LPS. In the RNA-seq data from another group analyzed here, the result is similar. By contrast, in all other species *CSF1* mRNA was expressed constitutively in BMDM. In our experience, by contrast to mouse, BMDM and MDM produced from other species including rat do not depend upon exogenous CSF1 for survival. This difference may be important in the interpretation of rodent models of the impact of human *CSF1R* mutations (Hume et al., 2020). BMDM from the different species differ also in their expression of other myeloid growth factors and receptors. *CSF2* (granulocyte macrophage CSF) was undetected in mouse but highly LPS-inducible in the ruminant and rat BMDM. *CSF3* was also LPS-inducible, especially in cow and rat BMDM. All the BMDM expressed the CSF2 receptor gene (*CSF2RA*) but only mouse BMDM expressed high levels of *CSF3R.*

One of the hallmarks of inflammation is the rapid recruitment of neutrophils in response to inducible chemoattractants. Some chemokine genes were expressed constitutively by BMDM, whilst others are major LPS target genes. Neutrophil recruitment in humans is mediated mainly by the CXC family of chemokines acting on neutrophil receptors CXCR1 and CXCR2 (Petri and Sanz, 2018). The most abundant neutrophil chemoattractant *CXCL8* (IL8) has no ortholog in rodents. The annotated *CXCL* chemokine genes detected in BMDM were either expressed constitutively, induced or repressed by LPS in individual species. In rodents in particular, neutrophil chemotaxis may also be mediated by the CC chemokine family acting through CCR1 (Petri and Sanz, 2018). The BMDM from large animals and rats shared high constitutive expression of *CCR1.* Orthology relationships amongst the CC chemokines were also ambiguous and most were not assigned in all species. Individual CC chemokine transcripts (*CCL1-CCL9*, *CCL11*, *CCL12*, *CCL14*, and *CCL17*) were expressed constitutively, induced or repressed in an idiosyncratic manner. The chemokine gene *CCL20*, which was not expressed in rodents but induced by LPS in humans and pigs (Schroder et al., 2012) was also expressed in all the ruminant BMDM. The small ruminants (sheep and goats) showed the unique LPS induction of another chemokine gene (*CCL24*) but in keeping with the apparent low interferon response expressed low levels of *CXCL10* (also known as interferon inducible peptide 10kD, or IP10). Chemokine receptors have been favored targets for anti-inflammatory drugs since their first discovery, and the relative failure of efforts to target them has been attributed to the fact that both ligands and receptors are promiscuous in their binding activity. A recent review summarizes evidence for non-redundant functions of multiple ligands binding to a shared receptor and vice versa (Dyer, 2020). However, chemokine redundancy would also provide an explanation for rapid evolutionary functional divergence, and it may be that each animal species has its own unique solution to the recruitment of inflammatory cells.

Members of the S100 family of cytoplasmic calcium binding proteins have many proposed functions as intracellular regulators and secreted mediators in macrophages (Xia et al., 2017). *S100A4*, *S100A10*, and *S100A11* were expressed constitutively in BMDM from all species. Ruminant BMDM share with pig BMDM constitutive high expression of *S100A8* and *S100A9* (which form a functional heterotetramer known as calprotectin). *S100A1* was restricted to mouse BMDM whereas *S100A12*, a likely duplication of *S100A8* that is absent from rodent genomes, was also highly expressed and induced further by LPS in BMDM from all the large animals (Supplementary Table S3) as it was in human MDM (Baillie et al., 2017). *S100A6* was expressed by rodents, pigs and horses, but absent from ruminant BMDM. Conversely, the ruminant BMDM were unique in expressing *S100B*. It is not clear whether any of these closely related S100 family members has a unique species-specific function or there is simply functional redundancy.

Where orthology relationships were unambiguous there are genes identified in the GCN analysis that appear extremely divergent amongst species. The most highly expressed transcript in all 4 ruminants, *SPP1* (encoding osteopontin, also known as secreted phosphoprotein 1), was expressed constitutively at a level around 10-fold higher than in rodents. The sheep (Clark et al., 2017) and pig (Summers et al., 2019) atlases reveal that *SPP1* is massively enriched in macrophages relative to all tissues and other cells, where this is not the case in mouse.^6^ *SPP1* is also amongst the most inducible transcripts in human MDM relative to blood monocytes (Baillie et al., 2017). There is a substantial literature on the many roles of osteopontin in innate immune regulation and mineral homeostasis, and genetic association with human disease susceptibility (Icer and Gezmen-Karadag, 2018). The numerous studies based upon the *Spp1* knockout mouse (Liaw et al., 1998) may not reveal the non-redundant functions of this gene in humans or large animals.

Manual curation reveals some examples where consistent expression is hidden by ambiguous annotation (e.g., *CD63*, annotated as the synonym *ITGA7* in the horse, is highly expressed in all species) and some examples (e.g., the absence of *CTSL* and *ATP6V0C* in both cattle and buffalo BMDM) where the lack of expression appears genuine. *CD163*, considered an M2 macrophage marker in mice (Murray et al., 2014) was expressed by BMDM from all the large animals but barely detected in mouse and rat. Horse BMDM showed uniquely high expression of *HSD11B1*, *IL5RA* and *TGFB3.* The inflammasome activator and cytoplasmic DNA sensor AIM2, known to be absent in pig and a pseudogene (low-expressed) in ruminants (Cridland et al., 2012) was LPS-inducible in horse BMDM. Genes within Cluster 14 that showed highest expression in the cow BMDM but were also highly expressed in other ruminants and pigs, include *ITGB3*, *MMP19*, *SLC31A2* (a copper transporter) and *VSIG4.* Genes within Cluster 10 that were most restricted to the buffalo include *CD34*, *CD247*, *CXCL2*, *IGF2*, *LDAH*, *LRR1*, *N2RF6*, *OSCAR*, and the zinc transporter *SLC39A2.* These transcripts could be associated with the adaptation of this species to tropical environments and high pathogen load.

## Conclusion

We have presented a network analysis of the response of BMDM from individual sheep to LPS providing strong evidence of a conserved feedback regulatory framework in mammalian macrophages. We then extended the analysis to compare the response across mammalian species. On balance, although Table 2 and the discussion highlight a number of clearly orthologous genes that show absolute expression differences among species, the number is small compared to the shared transcriptome and actually highlights the overall consistency of mammalian macrophage gene expression. We conclude that the vast majority of transcripts expressed constitutively or regulated by LPS in BMDM are detected in all species and most differences are quantitative rather than qualitative. In the analysis of multiple species we have used averaged data from outbred animals for every species other than *M. musculus* and rat. Based upon analysis of individuals in human, pig, and inbred mouse strains noted above, and the analysis of six crossbred sheep presented here (Figure 1), it is likely that there is also substantial qualitative and quantitative variation within each species. Such variation may ensure that populations display a variety of responses to the diversity of pathogen challenges, an evolutionarily advantageous strategy. On the other hand, the combined dataset provides a resource for the prioritization of candidate genes within loci associated with heritable differences in disease susceptibility in humans and livestock animals.

## Data Availability Statement

The datasets presented in this study can be found in online repositories. The names of the repositories and accession numbers can be found in Supplementary Table S1.

## Ethics Statement

All of the data analyzed herein were downloaded from publicly available datasets, generated independently in studies performed under appropriate Animal Ethics approvals, as detailed in the source publications.

## Author Contributions

SB, DH, and KS conceived the study, performed bioinformatic analysis, and wrote the manuscript. MM generated data for the sheep and performed bioinformatic analysis. EC and JP contributed to data generation and analysis and project supervision. ZL, CM, RY, and CP generated and contributed primary data for horse (ZL), goat and pig (CM), buffalo (RY), and rat (CP), respectively. All authors contributed to manuscript editing.

## Conflict of Interest

The authors declare that the research was conducted in the absence of any commercial or financial relationships that could be construed as a potential conflict of interest.

## Footnotes

1 https://github.com/lh3/seqtk

2 https://www.ensembl.org/info/genome/compara/Ortholog_qc_manual.html

3 http://biolayout.org

4 https://graphia.app

5 https://david.ncifcrf.gov/home.jsp

6 http://biogps.org

## Supplementary Material

FIGURE S1 Graph size vs. correlation threshold for sheep BMDM treated with LPS. The correlation threshold chosen was 0.75, which included 9,304 nodes making 2,569,091 edges.

FIGURE S2 Graph size vs. correlation threshold for nine species BMDM treated with LPS. The correlation threshold chosen was 0.8 (broken line), which included 8,129 nodes making 575,702 edges.
