# Supplementary material for: Species-Specificity of Transcriptional Regulation and the Response to Lipopolysaccharide in Mammalian Macrophages
Source: Front Cell Dev Biol. 2020 Jul 21;8:661. doi: 10.3389/fcell.2020.00661 (PMC7386301; doi:10.3389/fcell.2020.00661)
Supplement: FIGURE S1 — Graph size vs. correlation threshold for sheep BMDM treated with LPS. The correlation threshold chosen was 0.75, which included 9,304 nodes making 2,569,091 edges. [file Image_1.pdf]

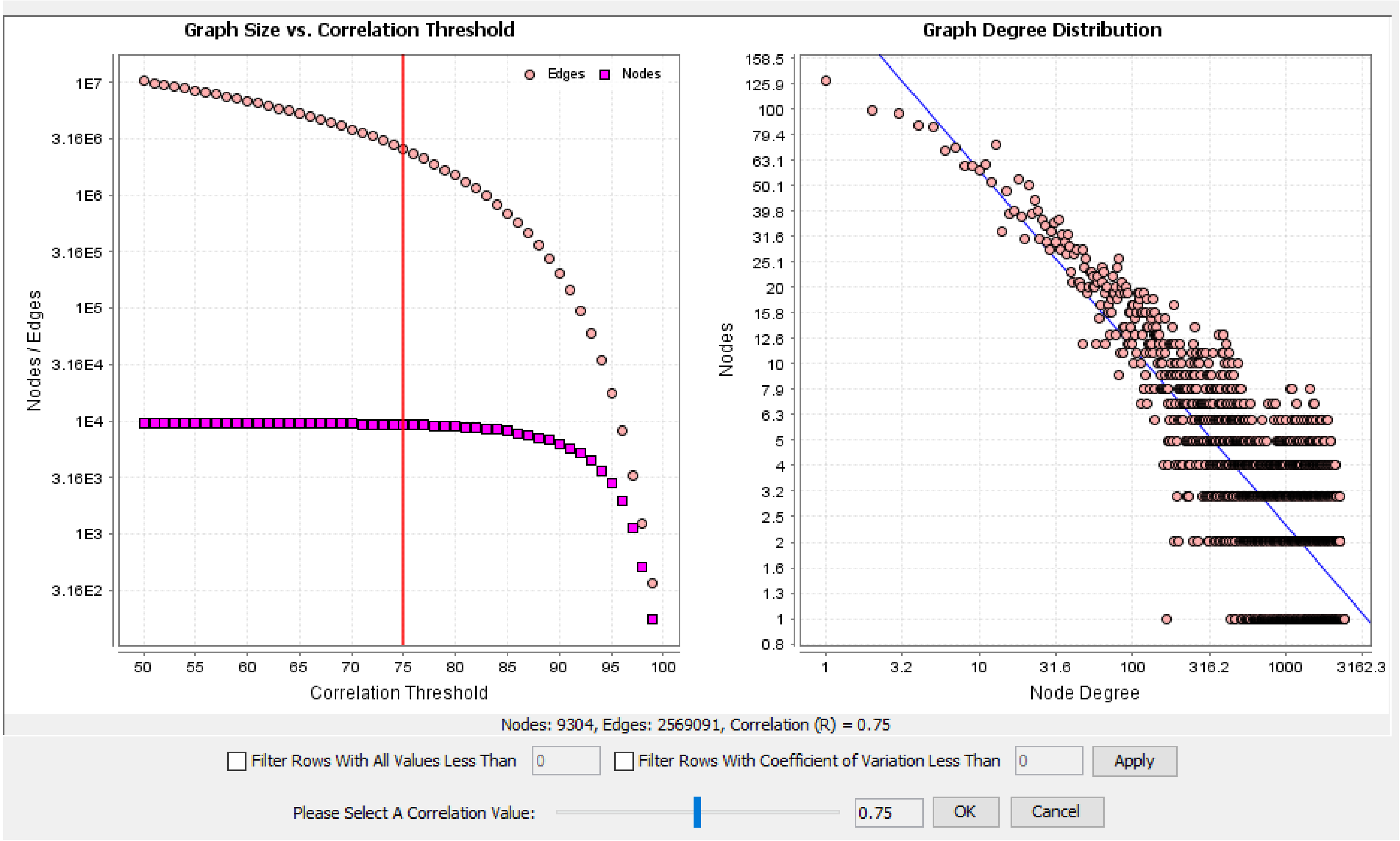

Supplementary Figure S1. Graph size vs correlation threshold for sheep BMDM treated with LPS. The correlation threshold chosen was 0.75, which included 9,304 nodes making 2,569,091 edges.
