# Supplementary material for: Species-Specificity of Transcriptional Regulation and the Response to Lipopolysaccharide in Mammalian Macrophages
Source: Front Cell Dev Biol. 2020 Jul 21;8:661. doi: 10.3389/fcell.2020.00661 (PMC7386301; doi:10.3389/fcell.2020.00661)
Supplement: FIGURE S2 — Graph size vs. correlation threshold for nine species BMDM treated with LPS. The correlation threshold chosen was 0.8 (broken line), which included 8,129 nodes making 575,702 edges. [file Image_2.pdf]

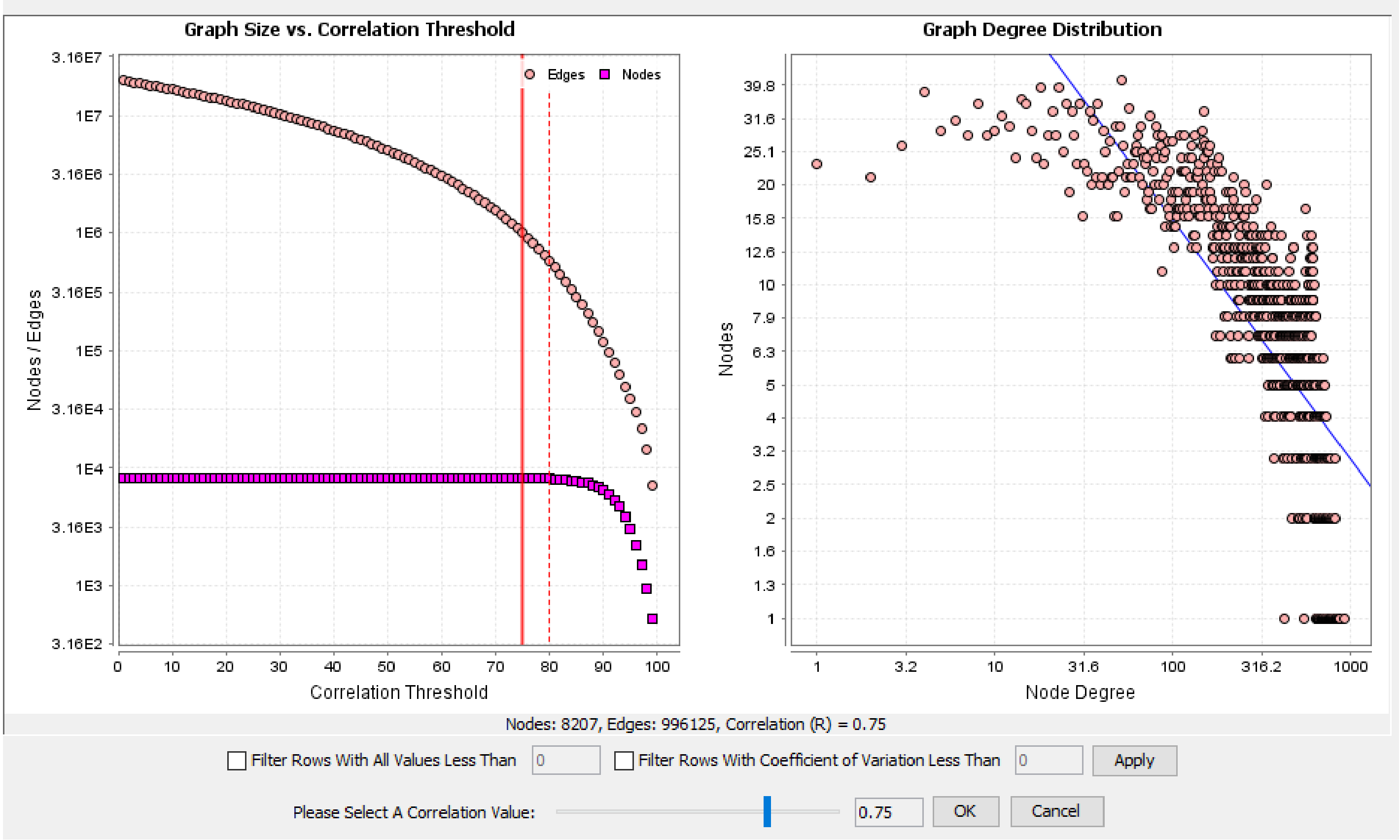


Supplementary Figure S2. Graph size vs correlation threshold for nine species BMDM treated with LPS. The correlation threshold chosen was 0.8 (broken line), which included 8,129 nodes making 575,702 edges.
